# Risk factors for irinotecan-induced hepatotoxicity: a retrospective cohort study

**DOI:** 10.3389/fonc.2026.1777922

**Published:** 2026-08-05

**Authors:** Fatemah A. Alherz, Anwar M. Alnakhli, Aisha M. Alqarni, Alanoud F. Alshammari, Alhanouf M. Ruwayi, Rahaf M. Alyamani, Haya Al-salloum, Mohammad J. Al-Yamani, Farag Shuweihdi

**Affiliations:** 1 Department of Pharmaceutical Sciences, College of Pharmacy, Princess Nourah bint Abdulrahman University, Riyadh, Saudi Arabia; 2 College of Pharmacy, Princess Nourah bint Abdulrahman University, Riyadh, Saudi Arabia; 3 College of Medicine, Almaarefa University, Riyadh, Saudi Arabia; 4 Department of Pharmacy Services, King Saud University Medical City, Riyadh, Saudi Arabia; 5 Department of Pharmacy Practice, College of Pharmacy, King Saud University, Riyadh, Saudi Arabia; 6 School of Dentistry, University of Leeds, Leeds, United Kingdom

**Keywords:** colorectal cancer, hepatotoxicity, irinotecan, irinotecan-induced hepatotoxicity, liver enzymes, liver function tests

## Abstract

**Background:**

Irinotecan is a potent chemotherapeutic agent used as a neoadjuvant in colorectal cancer liver metastasis and significantly enhances five-year survival rates. Recently, irinotecan-associated hepatotoxicity was a significant concern. Given the limited study in this area, we aimed to identify risk factors associated with irinotecan-induced hepatotoxicity.

**Methods:**

This is a retrospective cohort study at King Saud University Medical City in Riyadh. The study encompassed all adult cancer patients who received irinotecan treatment between Jun 2017 and December 2023. The clinical records of patients were reviewed to assess factors associated with hepatotoxicity. Time-to-event analyses were performed using time-varying exposure.

**Results:**

The study includes 229 adult cancer patients who received irinotecan-based treatment. Median age was 58 years, and colorectal cancer was the most diagnosed type of cancer (68.6%). Over a median follow-up of 103 days, the incidence of hepatotoxicity was 19.2% (n=44). In the adjusted analysis, higher baseline AST was associated with an increased hazard of hepatotoxicity (HR = 1.01; 95% CI: 1.01–1.02; *p* = 0.001). Cumulative irinotecan dose showed an inverse association with hepatotoxicity, with lower hazards observed in the medium cumulative dose group (HR = 0.36, 95% CI: 0.14–0.91, p = 0.031) and high cumulative dose group (HR = 0.06, 95% CI: 0.01–0.48, *p* = 0.008) compared with the low cumulative dose group.

**Conclusion:**

In summary, an inverse association was observed between cumulative irinotecan dose and hepatotoxicity. A higher baseline AST was also associated with hepatotoxicity, warranting careful assessment of pretreatment liver enzymes and closer monitoring during the early treatment period. Further studies are needed to validate these findings and investigate additional predictors of irinotecan-related hepatotoxicity.

## 1 Introduction

Irinotecan is a chemotherapeutic medication that inhibits DNA topoisomerase I, leading to DNA double-strand breaks and cell death (1). The FDA has recommended its use for colorectal cancer, and it is also used off-label for lung, ovarian, and gastric cancers (2, 3). Irinotecan has been shown to increase the five-year survival rate by around 58% when used as neoadjuvant chemotherapy in colorectal cancer liver metastasis (CRCLM) with unresectable tumors (4).

Irinotecan is a prodrug that is rapidly hydrolyzed by butyrylcholinesterase and carboxylesterase-1 and 2 to the active form 7-ethyl-10-hydroxycamptothecin (SN-38) (5, 6). SN-38 is then inactivated by cytochrome P450 (CYP) enzymes (CYP3A4 and CYP3A5) as well as uridine diphosphate glucuronosyltransferase 1A1 (UGT1A1) in the liver, resulting in the formation of inactive metabolites that are excreted in bile and urine (6). In the gastrointestinal tract, the hepatobiliary-excreted glucuronide metabolite of irinotecan can be converted back to the active form (SN-38) by bacterial β-glucuronidase, thereby prolonging irinotecan’s half-life and promoting its cytotoxicity (7).

Like other chemotherapeutic agents, irinotecan treatment is associated with adverse effects, some of which are serious, with the most reported toxicities being diarrhea and severe myelosuppression, which can lead to treatment failure or even death (3, 8). Furthermore, administration of irinotecan for a long duration or in high doses has been reported to impair liver parenchyma and liver regeneration, leading to hepatotoxicity, including hepatic steatosis or steatohepatitis (3, 4).

Irinotecan-induced hepatotoxicity is manifested mainly as increased transaminases, abnormal bilirubin, and nonalcoholic steatohepatitis, which have been observed to various degrees among people treated with irinotecan (3, 4, 9). The exact mechanism of irinotecan-induced hepatotoxicity has not been fully elucidated. However, mitochondrial dysfunction and autophagy have been proposed to be involved in irinotecan-induced steatohepatitis (10, 11).

The incidence of irinotecan-induced hepatotoxicity is especially elevated in patients with CRCLM, leading to higher morbidity and mortality rates (3, 4, 12). For instance, in a study of 153 patients with colorectal cancer liver metastasis receiving preoperative chemotherapy with either 5-fluorouracil, oxaliplatin, or irinotecan, hepatic injury was frequently found in patients treated with irinotecan. Out of 55 patients treated with irinotecan, 15 (27.3%) had hepatic steatosis, 2 (3.6%) had steatohepatitis, and 2 (3.6%) had grade 3 sinusoidal dilatation (13). In a study by Gómez-Ramírez et al., which includes 45 patients with colorectal cancer liver metastasis, 22 of them received irinotecan (n=7) or oxaliplatin (n=15) perioperatively, and 23 patients received no chemotherapy. Four out of 7 patients treated with irinotecan (57.2%) developed borderline or diagnostic steatohepatitis compared to the control group, and oxaliplatin with an incidence rate of 0% and 6.7%, respectively (12). In another study by Li et al. on 126 patients who received an irinotecan-based chemotherapy regimen, around 38.9% of the patients developed hepatotoxicity based on their biochemical liver function tests (3). Similarly, in a more recent retrospective multicenter cross-sectional study of 421 patients receiving irinotecan-based regimens, 92 patients (21.9%) developed liver injury as determined by biochemical liver function tests (14).

Few studies have identified risk factors associated with irinotecan-induced liver injury, including co-administration of platinum-based chemotherapy or CYP3A4 inducers, presence of liver metastasis, cardiovascular disease, alcohol consumption, cumulative dose or irinotecan ≥ 1000 mg, and three to four cycles of irinotecan (3, 4, 14).

The incidence of irinotecan-induced hepatotoxicity may influence treatment regimens by requiring a reduction in the dose or discontinuing the treatment, thus affecting cancer patients. Since irinotecan is influenced by several factors that contribute to interindividual drug response and toxicity, and to our knowledge, there is limited global data, and no available data on the factors contributing to irinotecan-induced hepatotoxicity in Saudi Arabia. This study aims to investigate potential risk factors associated with the development of irinotecan-induced hepatotoxicity in the Saudi population.

## 2 Methods

### 2.1 Study design/setting

The study was a retrospective cohort study conducted at the oncology center at King Saud University Medical City in Riyadh, Saudi Arabia. It aims to include all adult cancer patients who received irinotecan between Jun 2017 and December 2023. The study received approval from the Institutional Review Board of King Saud University in July 2023 (Project No. E-23-7930).

### 2.2 Study population and procedures

Cancer patients treated with irinotecan between Jun 2017 and December 2023 were admitted to the Oncology Center at King Saud University Medical City and were included in this study. This study included adults ≥18 years, cancer patients who received at least one dose of irinotecan-based chemotherapy, patients admitted between 2017 and 2023, and patients with an Eastern Cooperative Oncology Group score ≤2. This study excluded patients under 18 years old, who did not have a confirmed cancer diagnosis, or had been diagnosed with liver diseases (fatty liver, alcoholic liver cirrhosis, and hepatitis) before starting treatment. Additionally, patients were excluded if they had elevated aspartate aminotransferase (AST) or alanine aminotransferase (ALT) levels before treatment initiation. Liver enzyme elevation was defined as AST or ALT > 2 times the upper limit normal (ULN) in the absence of liver metastases, or more than 3 times ULN in the presence of liver metastases (15). Patients were also excluded if they had no baseline liver function tests (ALT, AST, and bilirubin) available prior to or after irinotecan treatment, or if they had uncontrolled comorbidities or an active infection.

### 2.3 Data collections

The demographic and clinical information, including age, body surface area (BSA), body mass index (BMI), gender, weight, smoking history, Eastern Cooperative Oncology Group status, tumor grade, underlying diseases such as diabetes or cardiovascular disease (CVD), chemotherapy regimens, and liver function test, including ALT, AST, Alkaline Phosphatase (ALP), and bilirubin. We included anticancer drugs, strong CYP3A inhibitors, and inducers, and UGT1A1 Inhibitors as concomitant medications. CYP3A4 inducers include phenytoin, phenobarbital, carbamazepine, and St. John’s wort, while CYP3A inhibitors include clarithromycin, indinavir, itraconazole, lopinavir, nefazodone, nelfinavir, ritonavir, saquinavir, telaprevir, and voriconazole) or UGT1A1 (e.g., atazanavir, gemfibrozil, indinavir) according to FDA drug labeling, since those medications may reduce or increase systemic exposure to irinotecan or its active metabolite (SN-38) (16, 17).

### 2.4 Medication administration and laboratory assessment

Patients who received an irinotecan monotherapy regimen or an irinotecan-based chemotherapy regimen at a minimum dose of 60 mg/m2 were included in the study. The classification of concomitant antineoplastic drugs in this study included antimetabolites (5-fluorouracil), programmed cell death protein 1 inhibitors (camrelizumab), monoclonal antibodies (cetuximab & bevacizumab), platinum-based chemotherapy (oxaliplatin & cisplatin), and molecular-targeted agents (apatinib). Liver function test data were collected prior to irinotecan administration and after 1 or 3 weeks after each cycle. Liver function tests were evaluated according to the guidelines of King Khalid University Hospital, with normal levels of ALT (16–61 units/L), AST (15–37 units/L), ALP (50–150 units/L), and total bilirubin (3–17 μmol/L). Hepatotoxicity was defined according to the National Cancer Institute Common Terminology Criteria for Adverse Events (CTCAE) v5.0. The CTCAE v5.0 defines grade I, grade II, grade III, and grade IV toxicity levels of transaminases (ALT and AST) as >1 to 3 x ULN if baseline was normal; >1.5–3 if baseline is abnormal, 3 to 5 x ULN if baseline was normal or abnormal, 5 to 20 x ULN if baseline was normal or abnormal, and >20 times the ULN if baseline was normal or abnormal, respectively. Total bilirubin was defined as grade I, grade II, grade III, and grade IV toxicity levels as >1 to 1.5 x ULN, >1.5 to 3.0 x ULN, 3.0 to 10.0 x ULN, and >10.0 x ULN, with normal or abnormal baseline, respectively. ALP was defined as grade I, grade II, grade III, and grade IV toxicity levels as > ULN - 2.5 x ULN if baseline was normal; 2.0 - 2.5 x baseline if baseline was abnormal, >2.5 - 5.0 x ULN if baseline was normal or abnormal, >5.0 - 20.0 x ULN if baseline was normal or abnormal, and >20.0 x ULN if baseline was normal or abnormal, respectively. In this study, we defined hepatotoxicity as one or more enzyme levels elevated and grade II or higher according to CTCAE.

### 2.5 Definition of cumulative irinotecan exposure

Exposure to irinotecan was measured as cumulative dose, defined as the sum of all administered doses for each patient from the start of treatment until one of the following events: hepatotoxicity, death, or the end of the study period, whichever occurred first. Cumulative irinotecan dose was categorized into three tertiles using the interval-level cumulative dose distribution. The low cumulative dose group ranged from 60 to 810 mg. The medium cumulative dose group ranged from 810 to 1,870 mg. The high cumulative dose group ranged from 1,870 to 8,450 mg.

### 2.6 Statistical analysis

All analyses were conducted using R software, version 4.6.1. Continuous variables were assessed for normality and summarized as medians with interquartile ranges (Q1–Q3). Between-group comparisons were performed using the Mann–Whitney test. Categorical variables were presented as frequencies and percentages and compared using the chi-square test or Fisher’s exact test, as appropriate.

Time-to-event analysis using Cox proportional hazards was performed to examine the association between cumulative irinotecan exposure and hepatic injury as the main outcome. Follow-up began at the start date of irinotecan treatment and ended at the end of irinotecan follow-up. Because irinotecan dose changed over time, the dataset was restructured into a start–stop survival format, with one record per patient per dose interval. For each interval, the cumulative irinotecan dose was calculated as the sum of all previous and current irinotecan doses up to that interval. Cumulative dose was then categorized into tertiles and modeled as a time-dependent covariate: low cumulative dose, medium cumulative dose, and high cumulative dose.

The fully adjusted model included cumulative dose group, liver metastasis, irinotecan-based monotherapy, previous cardiovascular disease, concomitant antimetabolite therapy, concomitant platinum therapy, concomitant monoclonal antibody therapy, baseline ALT, baseline AST, baseline bilirubin, age, sex, BMI category, cancer stage, and line of chemotherapy. Hazard ratios (HRs), 95% confidence intervals (CIs), and *p*-values.

The proportional hazards assumption was assessed for the adjusted Cox models using Schoenfeld residuals. There was no evidence of violation (*p*-value>0.05) of the model overall, based on the global test. Multicollinearity was assessed using generalized variance inflation factors. All adjusted GVIF values were low and close to 1. These values indicate that multicollinearity was not a concern in the adjusted model (data not shown).

## 3 Results

**Figure 1:**
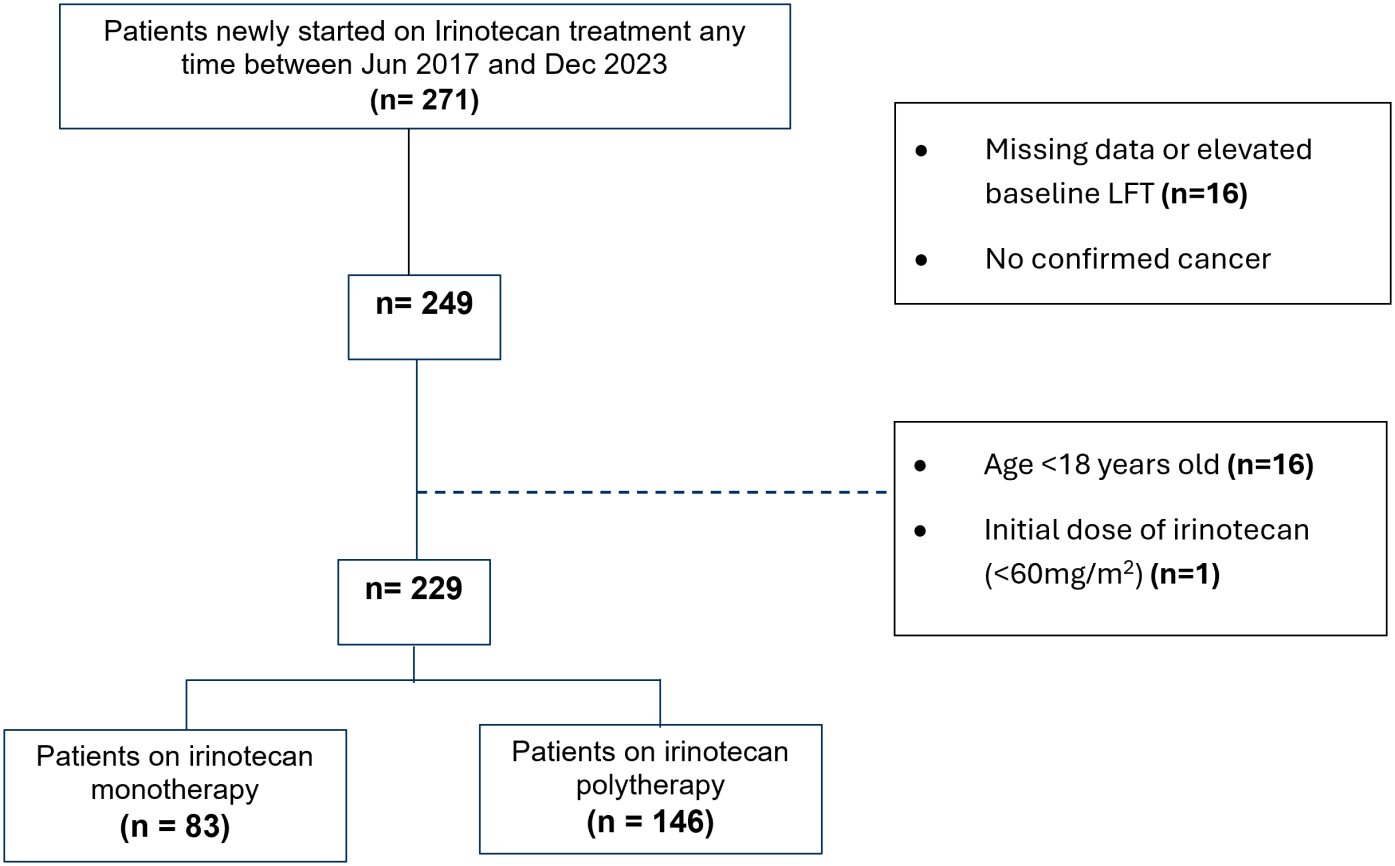
Flow chart of patient’s inclusion.

A total of 271 patients were screened between June 2017 and December 2023. After excluding ineligible cases, 229 patients were included in the final analysis (**Figure 1**). **Table 1** presents the demographic and baseline clinical characteristics of patients who received irinotecan therapy, stratified according to the presence or absence of the primary outcome. The median age was 58 years. Males were slightly more prevalent than females (56.5% vs. 43.5%) in the sample. Over half (57.2%) presented with stage IV disease, and 45.9% had liver metastases at baseline. The median recorded irinotecan dose was 285.57 (IQR 230-340), and the median final cumulative dose was 1127.5 mg (IQR 595-1984.25). Most patients were non-smokers (93.9%). Colorectal cancer was the most common (68.6%) diagnosed type of cancer, followed by pancreatic cancer (14.4%), brain cancer (8.3%), gastric cancer (3.5%), esophageal cancer (2.2%), and other cancer types (3.1%). Regarding chemotherapy treatment, 59% received irinotecan as second-line therapy, and 63.8% were treated with irinotecan in combination regimens. Common combined agents included antimetabolites (15.3%), platinum-based agents (11.4%), and monoclonal antibodies (32.8%). Comparison between patients with and without hepatotoxicity revealed no statistically significant differences in baseline characteristics, except for baseline AST level (*p* < 0.002) and presence of liver metastasis (*p* = 0.014), which differed significantly between the groups.

**Table 1:** Baseline characteristics of patients treated with irinotecan therapy, stratified by hepatic injury status.

Variable	All participants (n= 229)	Control group (n= 185)	Patients with hepatotoxicity (n= 44)	*P*-value
Age (years), median [IQR]	58.00 [50.00, 65.00]	58.00 [50.00, 66.00]	57.50 [48.75, 64.00]	0.641
BMI (Kg/m^2^), median [IQR]≤ 24 kg/m²	110 (48.0)	83 (44.9)	27 (61.4)	0.072
BMI (Kg/m^2^), median [IQR] >24 kg/m²	119 (52.0)	102 (55.1)	17 (38.6)	
Gender, n (%)				
Female	97 (42.4)	79 (42.7)	18 (40.9)	0.963
Male	132 (57.6)	106 (57.3)	26 (59.1)	
Smoking history, n (%)				
Yes	14 (6.1)	13 (7.0)	1 (2.3)	0.405
No	215 (93.9)	172 (93.0)	43 (97.7)	
Diabetes miletus, n (%)				
Yes	75 (33.6%)	37 (36.3%)	38 (31.4%)	0.495
No	75 (32.8)	63 (34.1)	12 (27.3)
Cardiovascular diseases, n(%)				
Yes	79 (34.5)	64 (34.6%)	15 (34.1%)	1.00
No	150 (65.5%)	121 (65.4%)	29 (65.9%)
Baseline liver enzyme level, median (IQR)				
ALT (U/L)	23.00 [16.00, 34.00]	22.00 [16.00, 33.00]	26.00 [17.75, 38.25]	0.236
AST (U/L)	21.00 [15.00, 32.00]	20.00 [14.50, 30.00]	27.00 [19.50, 38.25]	**0.002**
Total bilirubin (µmol/L)	7.30 [5.00, 10.15]	7.00 [5.00, 9.95]	9.13 [6.18, 12.95]	0.080
Type of diagnosed cancer, n (%)				
Colorectal cancer	157 (68.6)	124 (67.0)	33 (75.0)	0.333
Pancreatic cancer	33 (14.4)	25 (13.5)	8 (18.2)	
Brain Cancer	19 (8.3)	19 (10.3)	0 (0.0)	
Gastric cancer	8 (3.5)	7 (3.8)	1 (2.3)	
Esophageal cancer	5 (2.2)	4 (2.2)	1 (2.3)	
Other cancer	7 (3.1)	6 (3.2)	1 (2.3)	
Stage of Cancer, n (%)				
Stage I- III	98 (42.8)	81 (43.8)	17 (38.6)	0.652
Stage IV	131 (57.2)	104 (56.2)	27 (61.4)	
Liver Metastasis, n (%)				
Yes	105 (45.9)	77 (41.6)	28 (63.6)	**0.014**
No	124 (54.1)	108 (58.4)	16 (36.4)	
Line of chemotherapy, n (%)				
1^st^	20 (8.7)	16 (8.6)	4 (9.1)	0.909
2^nd^	135 (59.0)	108 (58.4)	27 (61.4)	
3^rd^ +	74 (32.3)	61 (33.0)	13 (29.5)	
Chemotherapy regimens, Irinotecan monotherapy n (%)				
Yes	83 (36.2)	64 (34.6)	19 (43.2)	0.373
No	146 (63.8)	121 (65.4)	25 (56.8)	
Antimetabolites (5-luorouracil and capecitabine), n(%)				
Yes	35 (15.3)	26 (14.1)	9 (20.5)	0.9408
No	194 (84.7)	159 (85.9)	35 (79.5)	
Platinum (cisplatin and oxaliplatin), n (%)				
Yes	26 (11.4)	20 (10.8)	6 (13.6)	0.79
No	203 (88.6)	165 (89.2)	38 (86.4)	
Monoclonal antibodies (bevacizumab, cetuximab, and trastuzumab), n (%)				
Yes	75 (32.8)	62 (33.5)	13 (29.5)	0.745
No	154 (67.2)	123 (66.5)	31 (70.5)	
Cumulative dose of irinotecan (mg), n (%)				
Low cumulative dose	77 (33.6)	59 (31.9)	18 (40.9)	0.373
Medium cumulative dose	76 (33.2)	65 (35.1)	11 (25.0)	
High cumulative dose	76 (33.2)	61 (33.0)	15 (34.1)	
Number of doses recorded, median [IQR].	6.00 [4.00, 10.00]	6.00 [4.00, 10.00]	5.50 [4.00, 8.00]	0.429
Final cumulative dose	1697.00 [1055.00, 2860.00]	1800.00 [1085.00, 2880.00]	1452.50 [947.75, 2785.00]	0.262
Final dose (mg), n(%)				
Low dose: <250 mg	80 (34.9)	64 (34.6)	16 (36.4)	0.697
Medium dose: 250–349 mg	102 (44.5)	81 (43.8)	21 (47.7)	
High dose: ≥350 mg	47 (20.5)	40 (21.6)	7 (15.9)	

ALT, alanine aminotransferase; AST, aspartate aminotransferase; BMI, body mass index; BSA, body surface area. Continuous variables are presented as median (IQR). Categorical variables are presented as n (%). Wilcoxon rank-sum tests were used for continuous variables, and chi-square tests were used for categorical variables.

Bold values indicate statistically significant p-values (p < 0.05).

Incidence of hepatotoxicity in this cohort was 19.2% (n=44). Regarding CYP3A4 inhibitors, only three patients were taking phenytoin, because of this very small number, CYP3A4-inhibitors use was not included in the multivariable model, as it would provide an imprecise statistical estimate.

The median follow-up time was 103 days, with the first quartile at 45 days. The median time from the initiation of irinotecan treatment to hepatic injury was 42 days (IQR 14–71 days). Kaplan–Meier analysis showed evidence of a difference in hepatic injury-free survival across final cumulative irinotecan dose groups. The log-rank test was statistically significant, χ² = 71.4, df = 2, *p* < 0.001.

**Figure 2:**
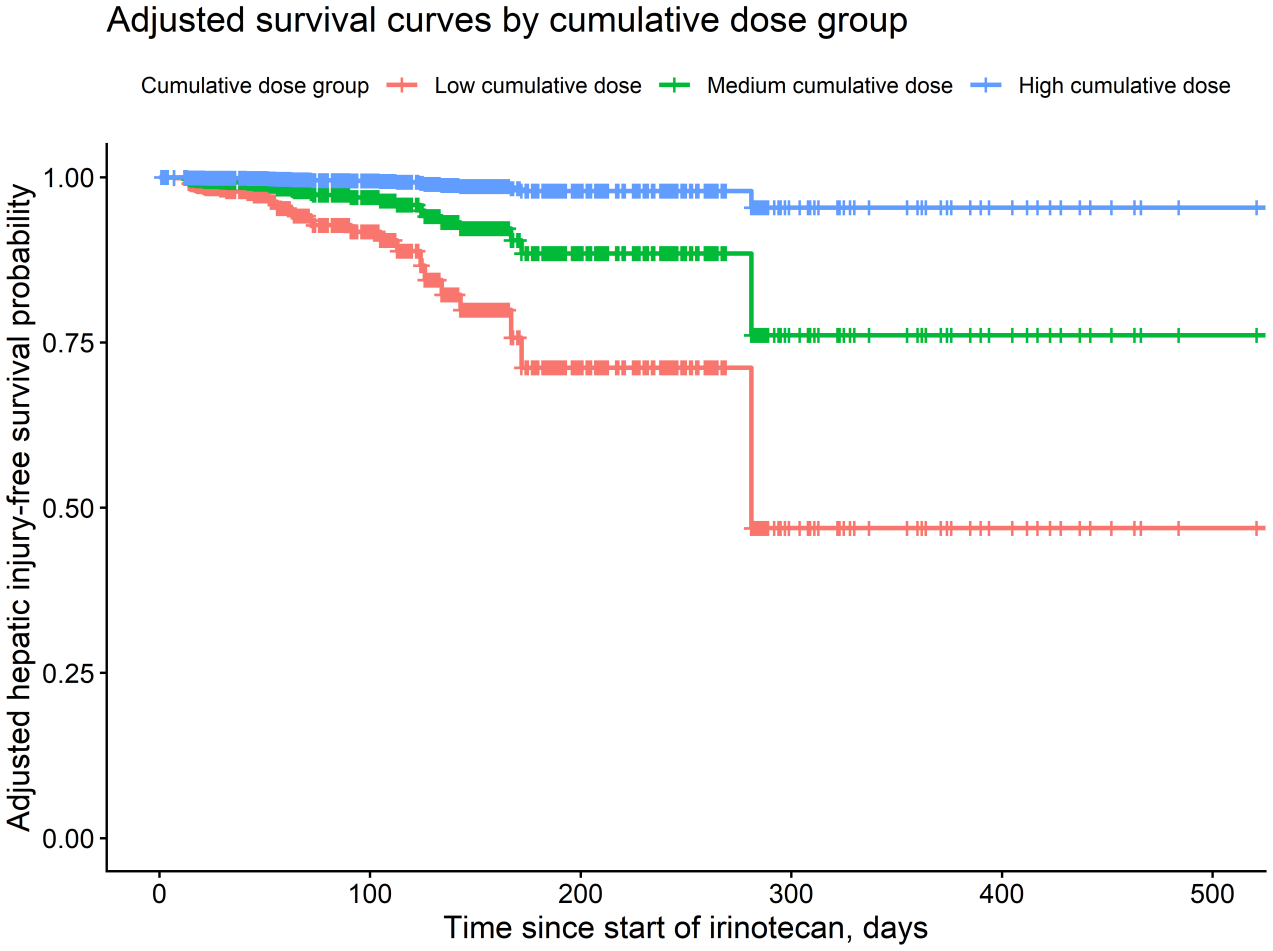
Adjusted survival plot for the onset of hepatotoxicity among patients treated with irinotecan in combination therapy. This plot shows the highest hazard for patients receiving lower cumulative irinotecan doses compared with those receiving higher cumulative doses or having more prolonged exposure.

In the adjusted model evaluating cumulative irinotecan dose, a higher cumulative dose was associated with a significantly lower hazard of hepatic injury (**Table 2**). Compared with the low cumulative dose group, the medium cumulative dose group had a reduced hazard of hepatic injury (HR = 0.36, 95% CI: 0.14–0.91, *p* = 0.031), while the high cumulative dose group had a markedly lower hazard (HR = 0.06, 95% CI: 0.01–0.48, *p* = 0.008). The adjusted survival curves showed separation between the cumulative dose groups, with the low cumulative dose group having the lowest probability of hepatic injury-free survival over time and the high cumulative dose group maintaining the highest probability (**Figure 2**). Baseline AST remained significantly associated with increased hazard of hepatic injury (HR = 1.01, 95% CI: 1.01–1.02, *p* = 0.001). Baseline ALT (*p* = 0.093) and baseline bilirubin (*p* = 0.085) showed borderline associations but did not reach conventional statistical significance.

**Table 2:** Adjusted Cox model for hepatic injury using cumulative dose group.

Predictor	HR	95% CI	*p*-value
Low cumulative dose			
Medium cumulative dose	0.36	0.14–0.91	**0.031**
High cumulative dose	0.06	0.01–0.48	**0.008**
Liver metastasis	1.77	0.92–3.43	0.090
Monotherapy before irinotecan	2.27	0.82–6.32	0.116
Previous cardiovascular disease	1.55	0.75–3.21	0.233
Previous antimetabolites	1.06	0.43–2.59	0.902
Previous platinum therapy	1.45	0.49–4.33	0.503
Previous monoclonal antibodies	1.64	0.65–4.13	0.299
Baseline ALT	0.99	0.98–1.00	0.093
Baseline AST	1.01	1.01–1.02	**0.001**
Baseline bilirubin	1.06	0.99–1.12	0.085
Age	0.99	0.96–1.02	0.489
Female sex	0.71	0.34–1.46	0.345
BMI (>24 kg/m²)	0.78	0.34–1.78	0.551
Cancer stage (IV)	1.40	0.71–2.76	0.337
Line 2 chemotherapy	1.07	0.35–3.23	0.907
Line 3+ chemotherapy	0.77	0.23–2.55	0.667

Reference group for cumulative dose: Low cumulative dose. *p*-values in bold indicate statistical significance at *p* < 0.05.

Hepatic outcomes of patients with hepatotoxicity are shown in **Table 3**. Irinotecan dose was reduced for 5 patients (11%), treatment was delayed for 7 patients (15.6%), and discontinued for 18 patients (40%) in response to elevated liver enzymes. No action was taken for 15 patients (33.3%). Most of them (n=12, 80%) had grade 2, while three patients (20%) had grade 3 hepatotoxicity. In addition, 9 patients have stage IV cancer (4 of them have liver metastasis), 2 patients have grade III and liver metastasis, 2 patients have grade II and liver metastasis, and one patient has grade I cancer. Ubon follow-up, 8 patients (53.3%) had progressive elevations of liver enzymes leading to treatment termination, after a few doses, and one patient had a treatment delay.

**Table 3:** Hepatic outcomes for patients with identified liver toxicity.

Hepatic outcome	N (%)
Dose reduction	5 (11%)
Treatment hold/delay for more than 30 days	7 (15.6%)
Treatment termination	18 (40%)
Grade 3 or greater severity	15 (33.3%) (grade 4(n=2), grade 3(n=13))

The total number of patients was 44. N, number.

## 4 Discussion

Colorectal cancer is the third most common cancer worldwide (18). In Saudi Arabia, it is the second most common cancer after breast cancer, with an incidence rate of 14.6% (19, 20). The FDA has approved irinotecan for treating colorectal cancer, yet its use has been challenged with serious adverse effects (3, 8). Recently, irinotecan-induced hepatotoxicity has been a growing concern since it might negatively impact patients with CRCLM (3, 4, 12). Given the incidence rate of colorectal cancer in Saudi Arabia and the fact that there is no data or study conducted in the country to investigate the risk factors associated with hepatotoxicity, this study was conducted to identify the risk factors associated with irinotecan-induced hepatotoxicity.

We conducted a retrospective cohort study of 229 cancer patients aged 18 years and older who received irinotecan in a single center in Saudi Arabia. The patients were mainly colorectal patients (68.6%). Our analysis reveals that hepatotoxicity is associated with baseline AST level and inversely associated with higher cumulative irinotecan exposure.

Previous research investigating irinotecan-induced hepatotoxicity did not adequately evaluate the role of baseline liver enzymes as predictors of hepatotoxicity (3, 14). However, the current study reveals that a 1-unit increase in the baseline serum AST level is associated with a 1% increase in the risk of hepatotoxicity (HR = 1.01, 95%CI= 1.01-1.02, *P* = 0.001). However, this increment might not translate directly into clinical practice. In a clinical setting, hepatic injury is rarely inferred from minor, single-unit fluctuations in serum enzyme levels, as these values naturally fluctuate within normal biological ranges. Rather, the clinical significance of liver enzyme elevation is assessed by the magnitude of increase relative to ULN, with hepatotoxicity graded according to the CTCAE criteria using the fold-increase value above ULN. Thus, such findings depend on the individual patient’s baseline and health condition.

Despite that, the finding of this study is similar to results from a study of patients with cancer treated with programmed cell death protein 1 inhibitors, which found that each 1-U/L increase in baseline AST was independently associated with a 12.5% increase in the odds of predominantly mild-to-moderate liver injury, defined as grades 1 and 2 (adjusted OR 1.125, 95% CI 1.035–1.224) (21).

Furthermore, although most patients in this study had liver enzyme levels within the normal range, this does not eliminate the risk of hepatotoxicity. According to the literature, the currently defined range of normal liver enzymes, particularly aminotransferase, has been reported in many studies to fail to identify people with well-defined liver diseases such as non-alcoholic steatohepatitis, autoimmune hepatitis, and chronic hepatitis C (22). In fact, according to a previous study, mortality is sometimes overlooked when aminotransferase values are below laboratory-set upper limits, which can lead to an increase in the mortality rate (23). For instance, a study reported that elevated or low liver enzyme levels (ALT and AST), even within the normal ranges, were found to be associated with a higher risk of mortality (24). The study also demonstrated a non-monotonic dose-response relationship between changes in liver enzymes over time and mortality, highlighting the complexity of translating the prognostic significance of hepatic biochemical abnormalities. Therefore, the study has suggested using changes in liver enzymes over time, as well as baseline levels, to predict mortality and assess risk by healthcare practitioners (24). This conclusion is also reinforced by medical agencies like the National Cancer Institute (NCI) Organ Dysfunction Working Group (ODWG) criteria, which are based solely on serum AST and total bilirubin levels to evaluate and categorize hepatic dysfunction and to guide dosing decisions in oncology patients, which points out the important role of evaluating AST to identify risk of liver injury (25, 26). Collectively, these findings suggest that close monitoring of liver function may be particularly important during the early phase of treatment, as early detection and timely intervention may prevent further deterioration of hepatic function.

Furthermore, in this study, we found that patients with higher and medium cumulative irinotecan doses had a lower hazard of haptic injury compared with those with low cumulative doses. Similar findings were reported in previous studies, in which a high cumulative dose of irinotecan was associated with the delayed onset of hepatotoxicity (3, 4). For instance, Han et al. reported a significant delay in the onset of hepatotoxicity with an irinotecan cumulative dose of 1000 mg or greater compared to patients receiving lower cumulative doses (14). This significant attribution may be due to the liver’s remarkable regenerative capacity, which can overcome drug-induced hepatotoxicity (27). For instance, a previous study by Takamoto et al. demonstrated that 2–4 weeks of chemotherapy cessation in patients receiving an irinotecan-based regimen (5-flurouracil, leucovorin, and irinotecan) improves hepatic functional reserve (28). Furthermore, it is important to point out that clinicians often reduce the dose, delay treatment, or discontinue chemotherapeutic agents based on the severity of liver injury (29), which prevents patients from receiving a higher cumulative dose. In fact, hepatic injury appeared to occur predominantly during the early phase of irinotecan treatment. In the present study, the median time from irinotecan initiation to hepatic injury was 42 days (IQR 14–71 days), comparable to the median onset of 46 days reported by Li J. et al. and 22 days reported by Furuse J. et al. Thus, an inverse association should be interpreted with caution, as patients who tolerate treatment for longer may be more likely to accumulate higher total doses, introducing potential survivor or treatment-tolerance bias.

Similar findings were also reported in previous studies evaluating the association of the number of irinotecan cycles and hepatotoxicity (3, 14). Han et al. reported a significant association with 3–4 cycles of irinotecan, whereas no significant association was reported in patients receiving five or more cycles (14). Likewise, Li et al. reported that an extended exposure period (more than 10 cycles) is associated with a reduced risk of developing hepatotoxicity compared to 5–9 cycles (3). Overall, although the liver has a regenerative capacity that could overcome drug-induced hepatotoxicity, compensatory liver regeneration can be inhibited if the drug dose is excessively increased (30). Therefore, clinicians should monitor liver enzymes closely and adjust the dose accordingly to prevent severe toxicity while maintaining effective cancer treatment.

In the present study, although 15 patients developed CTCAE grade 2 or 3 liver enzyme elevation, no immediate dose reduction or treatment modification was made at the time of toxicity. Most of these patients subsequently discontinue irinotecan after receiving a few doses because of further elevation in liver enzymes. This clinical management approach may reflect individualized risk-benefit considerations, given that the majority of these patients had advanced disease, with 60% presenting with stage IV cancer and an additional 13.3% with stage III and liver metastasis. A similar pattern was reported in a previous study of patients treated with nanoliposomal irinotecan-based therapy, in which grade 3 or 4 hepatic dysfunction was observed in 10 patients (21%). Of the reported patients, 1 had a dose reduction, 4 had dose interruption, and no action was reported for the remaining patients (31).

In contrast with previous studies by Han et al. and Li et al., which reported a significant contribution of liver metastasis in predicting irinotecan toxicity, with patients exhibiting 75% (OR = 1.75, 95%CI 1.08-2.93; *P=*0.023) and three-fold (OR = 3.51, 95%CI 1.71-7.18; *P* = 0.01) increased risk, respectively (3, 14), in this study no significant association was found with liver metastasis. The extent and severity of adverse effects of irinotecan in patients with CRCLM are related to the size of the liver lesion. Evidence from a large prospective cohort study indicates that irinotecan-related toxicities, including elevations in liver enzymes, were more likely to occur in patients with larger metastatic lesions replacing normal hepatic tissue (32). However, the current study, as well as previous studies investigating liver metastases as a risk factor for hepatotoxicity, did not include data on the extent of hepatic substitution by metastatic lesions (3, 14). Therefore, the degree of preexisting hepatocellular damage could not be quantified. This limitation may therefore lead to an overestimation or underestimation of the true association between liver metastases and hepatotoxicity, which could explain the inter-patient variability in hepatotoxicity risk observed across studies. Finally, compared with previous studies reporting significant associations between irinotecan-induced hepatotoxicity and co-administration of platinum-based chemotherapy or CYP3A4 inducers, alcohol consumption, and cardiovascular diseases, this study did not find significant associations between these factors and irinotecan-induced hepatotoxicity (3, 14). This might be due to differences in the criteria used to evaluate hepatotoxicity, in the study population, or in sample size. Furthermore, in this study, we could not evaluate the association between alcohol consumption and hepatotoxicity because patients’ records lacked information about alcohol consumption. In addition, with respect to CYP3A4-inducing medications, only three patients were taking phenytoin at baseline, making it impossible to evaluate the effect of CYP3A4 inducers on the association with irinotecan-induced hepatotoxicity.

In clinical practice, irinotecan dosing is traditionally guided by its well-established dose-limiting toxicities (DLTs)—including diarrhea and neutropenia. These adverse effects have consistently been shown to increase with higher irinotecan exposure and have shaped current dosing strategies. The findings of our study suggest that the relationship between irinotecan and liver injury is more likely mediated by the patient’s underlying clinical condition. In fact, a previous study suggested evaluating patients for metabolic syndrome-related risk factors, including hypertension, diabetes, obesity, and hyperlipidemia, before initiating irinotecan therapy (33). Thus, close monitoring of liver enzymes during treatment is crucial for promptly detecting hepatic adverse effects and addressing potential adverse events, ensuring patient safety and optimizing therapeutic outcomes.

Furthermore, predefining patient factors related to liver function and selecting the optimal initial dose for each patient to reduce the chance of future complications, improve patient tolerability, and maintain the patients in the treatment regimen for a longer period. In fact, Raymond et al. showed that patients with elevated bilirubin levels of 1.51–3 times the ULN who received reduced-dose irinotecan (200 mg/m^2^) tolerated the treatment for a longer period (10 cycles) without dose reduction or treatment delay.

The strengths of this study include the ability to follow patients from initiation of irinotecan therapy through the development of hepatotoxicity, thereby enabling a more precise assessment of exposure and outcome. The cumulative irinotecan dose was modeled as a time-varying covariate, allowing exposure to be updated during follow-up and aligned with the corresponding risk period. Furthermore, the use of a rigorous definition of hepatotoxicity minimized the risk of outcome misclassification. Importantly, this is the first study in Saudi Arabia to examine risk factors associated with irinotecan-induced hepatotoxicity.

However, this study had several limitations that may account for its modest findings regarding factors associated with irinotecan-induced hepatotoxicity in cancer patients. One limitation is that our study was conducted at a single center and had a relatively small sample size of 229 patients, which may hinder the detection of statistically significant differences. In addition, this study excluded patients with preexisting liver diseases, which may limit its generalization to patients with chronic liver disease. Evaluation of hepatotoxicity was based solely on liver enzyme test; thus, autoimmune hepatitis and irinotecan-induced steatohepatitis might be overlooked due to the invasive nature of liver biopsy, which serves as the gold standard for diagnosis and can only be conducted on a small fraction of patients. Additionally, the type and severity of toxicity were not consistently documented in medical records, leaving a gap for further analysis. Given these limitations, a prospective multicenter study involving a larger, more diverse patient population is recommended. Additionally, evaluation of the number of irinotecan cycles, gamma-glutamyl transferase, International Normalized Ratio (INR), and albumin levels to assess liver function; degree of hepatic substitution in case of liver metastasis; and a history of liver resection surgery or pre-existing hepatic disorder and steatotic liver disease. These would thereby improve the study’s statistical power and yield more comprehensive insights into risk factors for irinotecan-induced hepatotoxicity.

## 5 Conclusion

In summary, our study revealed that AST level and cumulative irinotecan dose were the most significant factors associated with hepatotoxicity in patients receiving irinotecan. These findings emphasize the need to identify and monitor these risk factors to optimize patient care and treatment outcomes in cancer management protocols involving irinotecan.

## Data Availability

The raw data supporting the conclusions of this article will be made available by the authors, without undue reservation.
